# Procalcitonin, IL-10 and sCD25 as diagnostic and prognostic markers in critically ill patients

**DOI:** 10.1186/cc11730

**Published:** 2012-11-14

**Authors:** A Focà, MC Liberto, L Rametti, A Giancotti, N Marascio, A Quirino, S Caroleo, A Renzulli, G Matera

**Affiliations:** 1 University of Catanzaro Institute of Microbiology, Catanzaro, Italy; 2 University of Catanzaro Anesthesiology Unit, Catanzaro, Italy; 3 University of Catanzaro Cardiac Surgery Unit, Catanzaro, Italy

## Background

Diagnostic and prognostic markers are of outmost importance in the critical patient referred to the ICU. The aim of the present study was the evaluation of procalcitonin (PCT), IL-10 and soluble CD25 (sCD25) as potential markers in the diagnosis and prognosis of cohorts of critical patients evaluated following a prospective design in a tertiary-center university hospital.

## Methods

We studied 52 consecutive SIRS patients with suspected sepsis that were firstly stratified based on culture results (culture-positive and culture-negative), then subsequently divided into survivors and nonsurvivors. Venous blood samples were obtained from each patient within 6 hours of hospital/ICU admission (T-0) and at the end of first week of hospital/ICU stay (T-1). Serum samples were collected and used for simultaneous determination of IL-10 and sCD25, using a cytokine biochip array on the Evidence Investigator analyser (Randox Laboratories Ltd, Crumlin, UK), while PCT was assayed by an enzyme-linked fluorescent assay (VIDAS BRAHMS PCT; bioMerieux, France). Statistically significant differences between groups were established by the Mann-Whitney U test. The receiver-operating characteristic (ROC) curve was used to evaluate the diagnostic accuracy (defined by the area under the ROC curve (AUROCC)) of the analyzed cytokines and to determine the sensitivity and specificity at selected cutoff values. The statistical analyses were performed using SPSS 14.0 software (SPSS, Chicago, IL, USA).

## Results

PCT, IL-10 and sCD25 were significantly (*P *< 0.05) increased in infectious versus non-infectious SIRS patients at hospital admission (T-0) and after 1 week of hospital stay (T-1). Diagnostic accuracy of PCT, IL-10 and sCD25 was evaluated by AUROCC and exhibited a significance index of 0.0001, 0.0021 and 0.0095 respectively at T-0, while at T-1 the *P *values were 0.0011, 0.0016 and 0.0201 respectively. Regarding prognostic markers, PCT showed a prognostic significance only at T-1 (*P *= 0.04). However when IL-10 and sCD25 values from survivors were compared with those from nonsurvivors, significant differences were found for the former and the latter marker with *P *= 0.0014 and *P *= 0.014 respectively at T-0, as well as with *P *= 0.0002 and *P *= 0.014 respectively at T-1.

## Conclusion

In our study, well-known PCT and IL-10 markers and novel sCD25 significantly contributed to diagnosis and prognosis of SIRS and septic patients.

